# Artesunate shows potent anti-tumor activity in B-cell lymphoma

**DOI:** 10.1186/s13045-018-0561-0

**Published:** 2018-02-20

**Authors:** Thea Kristin Våtsveen, Marit Renée Myhre, Chloé Beate Steen, Sébastien Wälchli, Ole Christian Lingjærde, Baoyan Bai, Pierre Dillard, Theodossis A. Theodossiou, Toril Holien, Anders Sundan, Else Marit Inderberg, Erlend B. Smeland, June Helen Myklebust, Morten P. Oksvold

**Affiliations:** 1 Department of Cancer Immunology, Institute for Cancer Research, Oslo University Hospital-Radiumhospitalet, Ullernschausseen 70, Montebello, 0379 Oslo, Norway; 2 Centre for Cancer Biomedicine, University of Oslo, Oslo, Norway; 3 Department of Cellular Therapy, Department of Oncology, Oslo University Hospital-Radiumhospitalet, Oslo, Norway; 4 Department of Computer Science, University of Oslo, Oslo, Norway; 5 Department of Radiation Biology, Institute for Cancer Research, Oslo University Hospital-Radiumhospitalet, Oslo, Norway; 6 Department of Clinical and Molecular Medicine, Faculty of Medicine and Health Sciences, Norwegian University of Science and Technology, Trondheim, Norway; 7 Department of Hematology, St. Olav’s Hospital HF, Trondheim, Norway

**Keywords:** Artemisinin, B-cell lymphoma, Drug screen, ER stress, UPR

## Abstract

**Background:**

Although chemo-immunotherapy has led to an improved overall survival for most B-cell lymphoma types, relapsed and refractory disease remains a challenge. The malaria drug artesunate has previously been identified as a growth suppressor in some cancer types and was tested as a new treatment option in B-cell lymphoma.

**Methods:**

We included artesunate in a cancer sensitivity drug screen in B lymphoma cell lines. The preclinical properties of artesunate was tested as single agent in vitro in 18 B-cell lymphoma cell lines representing different histologies and in vivo in an aggressive B-cell lymphoma xenograft model, using NSG mice. Artesunate-treated B lymphoma cell lines were analyzed by functional assays, gene expression profiling, and protein expression to identify the mechanism of action.

**Results:**

Drug screening identified artesunate as a highly potent anti-lymphoma drug*.* Artesunate induced potent growth suppression in most B lymphoma cells with an IC_50_ comparable to concentrations measured in serum from artesunate-treated malaria patients, while leaving normal B-cells unaffected. Artesunate markedly inhibited highly aggressive tumor growth in a xenograft model. Gene expression analysis identified endoplasmic reticulum (ER) stress and the unfolded protein response as the most affected pathways and artesunate-induced expression of the ER stress markers ATF-4 and DDIT3 was specifically upregulated in malignant B-cells, but not in normal B-cells. In addition, artesunate significantly suppressed the overall cell metabolism, affecting both respiration and glycolysis.

**Conclusions:**

Artesunate demonstrated potent apoptosis-inducing effects across a broad range of B-cell lymphoma cell lines in vitro, and a prominent anti-lymphoma activity in vivo, suggesting it to be a relevant drug for treatment of B-cell lymphoma.

**Electronic supplementary material:**

The online version of this article (10.1186/s13045-018-0561-0) contains supplementary material, which is available to authorized users.

## Background

More than 60 different B-cell lymphoma types have been described, with different clinical courses and outcomes [1]. Diffuse large B-cell lymphoma (DLBCL) and follicular lymphoma (FL) are the most common forms of aggressive non-Hodgkin lymphoma (NHL) and indolent NHL, respectively. Current standard of care often involves chemotherapy regimens combined with monoclonal antibodies targeting the B-cell surface molecule CD20 (rituximab) and has led to an improved overall survival for most lymphoma types [2]. A number of small molecule inhibitors are currently under clinical testing, where the majority represents inhibitors of the B-cell receptor signaling (BCR) pathway and downstream signaling molecules [3].

Artesunate is a semisynthetic analogue of artemisinin, extracted from the sweet wormwood plant, *Artemisia annua*, and is the first-line treatment of severe malaria [4]. Artemisinins have demonstrated cytotoxic effects in vitro against a range of cell lines and also to be effective against drug-resistant cancer cell lines [5, 6], although the underpinning mechanisms remain unclear [7, 8]. Few studies have been reported regarding clinical use of artesunate in cancer, but artesunate has demonstrated anti-tumor effects in a small randomized clinical trial in colon cancer and to transiently decrease tumor size and prostate-specific antigen levels in a patient with advanced prostate cancer [9, 10]. The supply of artesunate has been limited, but semisynthetic production since 2014 has increased the availability [11] and enables additional clinical use of artesunate beyond malaria treatment.

B lymphoma cell lines are representative models with > 80% match with the corresponding primary cancer cells [12, 13] (O. Kallioniemi, pers. com.). Here, we demonstrate a potent induction of apoptosis by artesunate in a broad range of cell lines, representing the most common types of B-cell lymphoma. Artesunate also showed potent anti-tumor efficacy in vivo in a xenograft model, providing a rationale for clinical testing as B-cell lymphoma therapy.

## Methods

### Materials

Small molecule inhibitors are as follows: everolimus, dasatinib, duvelisib, alisertib, ibrutinib, idelalisib, sorafenib, metformin, and entospletinib (Selleckchem, Houston, TX; further information in Additional file 1: Table S1). All chemicals were from Sigma-Aldrich (St. Louis, MD) unless otherwise noted. Primary antibodies are as follows: rabbit anti-ATF-4 (#11815), -ATF-6 (#65880) and –β-tubulin (#2128) and mouse-anti-DDIT3/CHOP (#2895) (Cell Signaling Technology, Danvers, MA), rabbit anti-GAPDH (#100118; GeneTex, Irvine, CA), mouse anti-HSP70 (#648001; BioLegend, San Diego, CA), goat anti-Lamin-B (#SC-6217; Santa Cruz Biotechnology, Dallas, TX). Secondary antibodies are as follows: HRP-conjugated goat anti-rabbit (#111-035), goat anti-mouse (#115-035), and donkey anti-goat (#705-035) IgG antibodies (Jackson Immunoresearch, West Grove, PA).

### Cell lines, human samples, and culture conditions

Cell lines representing the most common B-cell lymphoma subtypes are the following: Burkitt lymphoma: BL-41, Raji, Ramos, Rec-1 (Leibniz-Institut-Deutche Sammlung von Mikroorganismen und Zellkulturen (DSMZ)) and Namalwa (gift from J. Delabie); Germinal Center B-like (GCB) DLBCL: ULA, SU-DHL-6, Oci-Ly-18 (DSMZ), Oci-Ly-2, SU-DHL-4 (gift from L. Staudt); activated B-cell like (ABC) DLBCL: U-2932 (DSMZ); DLBCL-double hit: Will-2 (DSMZ); mantle cell lymphoma: Mino, JeKo-1, Granta-519 (DSMZ); FL: Ros-50 (DSMZ), K-422 (gift from J. Delabie); immunoblastic B-cell lymphoma: DoHH-2 (DSMZ). K-422, Oci-Ly-2, Namalwa and Ros-50 were authenticated by STR-profile in 2011, BL-41, SU-DHL-4, SU-DHL-6, Ramos, Raji, Granta, WILL-2, Rec-1. Mino and DoHH-2 were DNA fingerprinted (December 2017, Forensik, Eurofins Medigenomix, Ebersberg, Germany). The cell lines obtained as gifts were second generation passages. For routine use of the cell lines, second generation splits were thawed and the experiments were performed in the time frame of 1 week after thawing and to maximum 20 passages. Mycoplasma testing is always performed after 4 weeks for all cultures using PCR-based methods (Venor GeM Mycoplasma Detection Kit), and always prior to injection of cells into mice. Cell lines were cultured in RPMI-1640 supplemented with 10% fetal calf serum (FCS) or 10% human serum (HS; TCS Biosciences, Buckingham, UK), penicillin, and streptomycin (complete media) and maintained at 37 °C in 5% CO_2_. B-cells were isolated from buffy coats from peripheral blood from anonymous, healthy donors at the Blood Bank (Oslo University Hospital, Norway), after informed consent and approval from regional authorities (REK S-03280). B-cells were isolated using anti-CD19 Dynabeads (Thermo Fisher Scientific, Waltham, MA), followed by the corresponding DETACHaBEADs [14]. B-cells were maintained in complete media with rhCD40 ligand (CD40L) (0.25 μg/ml; Enzo Life Sciences, Farmingdale, NY) preincubated for 30 min with an enhancer for ligands (1 μg/ml; Enzo Life Sciences) and recombinant human interleukin (IL)21 (50 ng/ml; Thermo Fisher Scientific).

### Luciferase construct and BL-41-luc cell line

The gene encoding for luciferase-TropC-GFP [15] was subcloned into pENTR vector and further recombined into pMP71 retroviral vector [16]. Retroviral particles were prepared, and BL-41 cells were transduced by double spinoculation [17]. Positive cells were sorted for GFP.

### Viability and apoptosis assays

Cells were grown in 96-well plates (10,000 cells/well) for 72 h with or without artesunate (0.125–8 μM) and small molecular inhibitors (Additional file 1: Table S1). The CellTiterGlo Assay (Promega, Madison, WI) was used for measuring relative cell growth (ATP levels), using Modulus microplate reader (Turner Biosystems, Promega). Measurements were related to control and reported as relative luminescence units (RLU). Viability was measured by propidium iodide (PI) assay (1 μg/ml; Thermo Fisher Scientific) after treatment with artesunate (5–10 μM) for 72 h. Apoptosis was detected by active caspase-3 assay (Alexa-647-rabbit anti-active caspase-3 clone C92-605; BD Biosciences, CA) after 24 h treatment with artesunate (1 and 5 μM) with and without Z-VAD-FMK (1 μl/ml (ab65613), Abcam, Cambridge, UK). Terminal deoxynucleotidyl transferase dUTP nick end labeling (TUNEL) was determined by the In Situ Cell Death Detection Kit after 72 h treatment with artesunate (10 μM). For both apoptosis assays, cells were fixed in PFA for 5 min and permeabilized with ice cold methanol. All assays were analyzed using FACS Canto II or LSR II flow cytometer (BD). Flow cytometry data were analyzed using the online Cytobank flow cytometry software (www.cytobank.org) [18]. DL-buthionine sulfoximine (BSO) was used at 50 μM for 18–24 h for reducing the cellular glutathione levels by inhibiting γ-glutamylcysteine synthetase. Glutathione levels were measured by GSH-Glo Glutathione assay (Promega).

### Gene expression profiling

Total RNA was isolated using MiRNeasy (Qiagen, Hilden, Germany) after 4 and 12 h treatment with artesunate (5 μM). The microarray analyses were performed on Illumina’s HumanHT-12 v4 Expression BeadChip platform (Illumina, San Diego, CA). The differential gene expression analysis was done using the *limma* package [19] in R (version 3.3.1). Two technical replicates were run per cell line and time point, and the results were averaged together. The differentially expressed genes were selected based on a log fold change larger than the absolute value of 0.5, and an adjusted *p* value (FDR) of less than 0.01. The probes were collapsed according to gene symbol, using the annotation file for the Illumina’s HumanHT-12 v4 Expression BeadChip platform. When several probes mapped to the same gene, the probe with lowest log fold change was selected. The pathways and networks most enriched for the differential expressed genes were identified by Ingenuity Pathway Analysis (IPA) software (Qiagen) with default settings. Microarray data is available at NCBI’s Gene Expression Omnibus with accession number GSE94553 (https://www.ncbi.nlm.nih.gov/geo/query/acc.cgi?acc=GSE94553).

### Immunoblotting

Cells were lysed and processed for SDS-PAGE [20]. Miniprotean or Criterion TGX precast gels were used for SDS-PAGE (Bio-Rad Laboratories, Hercules, CA). SuperSignal West Pico or Dura (Thermo Fisher Scientific) or Clarity (Bio-Rad) was used for detection. Chemidoc MP (Bio-Rad) was applied for imaging, and image processing was performed in ImageLab (Bio-Rad), Adobe Photoshop, and Adobe Illustrator (Adobe Systems, San Jose, CA).

### Animal experiments

The care and handling of animals for the present study were in conformity with the Norwegian Food Safety Authority in compliance with the European Convention of the Protection of Vertebrates Used for Scientific Purposes (Project ID 7729). NOD.Cg-Prkdc^scid^ Il2rg^tm1Wjl^/SzJ (NSG) mice were bred in-house. Pilot experiments were performed with three mice in each group. Based on the results, *n* = 10 for treatment and control group was chosen. The mice (6–10 weeks old) were injected subcutaneously with 2 × 10^6^ BL-41 cells expressing firefly luciferase (BL-41-luc). Tumor take was measured by IVIS at day 4 before mice were divided into control and treatment groups. The mice were divided according to tumor size within each cage for either treatment or control group to have non-biased, comparable groups. Artesunate was dissolved in EtOH/DMSO (1:1) to 370 mg/ml and diluted 1:10 in 5% Na_3_CO_3_ before injections. Mice were injected daily from day 4 with 200 mg/kg artesunate intraperitoneally or control (5% EtOH and 5% DMSO in Na_3_CO_3_). Treatment was given for 12 days, then 2 days off, followed by treatment every day until day 19 (17 injections total). Tumor growth was monitored by bioluminescent imaging (IVIS spectrum in vivo imaging system) at regular intervals. Mice were injected intraperitoneally with 150 mg/kg d-luciferin (Caliper Life Sciences, Hopkinton, MA) and imaged after 10 min. Inhalation anesthetic sevofluran (Baxter) supplemented with O_2_ and N_2_O was used during imaging. Caliper measurement was used to determine if the tumor size had reached maximum 2 cm in one direction or 2 cm^3^ that was the limit for euthanasia.

### Seahorse assay

A Seahorse Extracellular Flux (XF96e) Analyzer (Agilent, Santa Clara, CA) was used to measure the oxygen consumption rate (OCR) which relates to mitochondrial respiration and the extracellular acidification rate (ECAR) reflecting the cellular glycolytic activity of live intact B-cell lymphoma cells, in real time with and without artesunate pretreatment (5 μM, 12 h). Prior to the assay, BL-41, SU-DHL-6, and Mino cells were attached onto Cell-Tak (Corning Inc., Corning, NY) coated 96-well XF-PS plates at a density of 1 × 10^5^ cells/well in DMEM XF unbuffered assay media, supplemented with 2 mM sodium pyruvate, 10 mM glucose, 2 mM l-glutamine and adjusted to physiological pH (7.6) and incubated in the absence of CO_2_ for 1 h prior to the seahorse measurements (*n* = 21 technical replicates). Initially, the cell basal respiration was measured for all groups while after injection of oligomycin, a potent F1F0 ATPase inhibitor (1 μM), the OCR dropped by the amount required for ATP production by respiration. Subsequent addition of 1 μM carbonyl cyanide p-trifluoromethoxyphenylhydrazon (FCCP) uncoupled the mitochondrial electron transport from ATP synthesis and hence revealed the maximal respiration capacity in each case. Finally, addition of 1 μM antimycin A and rotenone completely inhibited the electron transport and hence respiration. Initially, the basal glycolytic rates of the treatment groups were measured while upon addition of oligomycin to the cells, being unable to produce ATP by respiration switched to their highest glycolytic capacity to compensate for their ATP requirements.

### Statistical testing

Statistical significance was determined by two-tailed unpaired Student’s *t* test and two-tailed heteroscedastic Student’s *t* test for Seahorse assay. One-sided *t* test was used for animal studies, in addition to log-rank test for the Kaplan-Meier plots. GraphPad Prism and Igor Pro were used for calculations. Differences were considered to be statistically significant if *P* < .05.

## Results

### Artesunate has potent anti-lymphoma activity in vitro

**Fig. 1:**
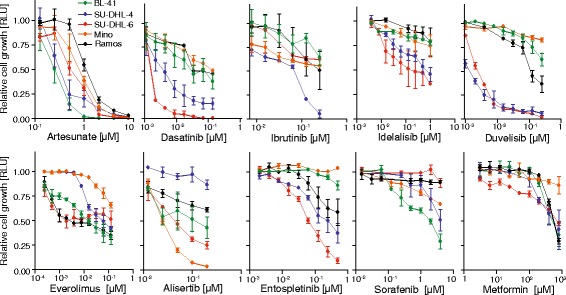
Potent growth suppression by artesunate in B-cell lymphoma cell lines. The dose-response effect for each drug was tested within a clinically relevant concentration range, and cell growth was measured using CellTiterGlo following 72 h treatment. Relative luminescence units (RLU) compared to untreated cells is shown (mean ± SD, *n* = 2–4)

**Fig. 2:**
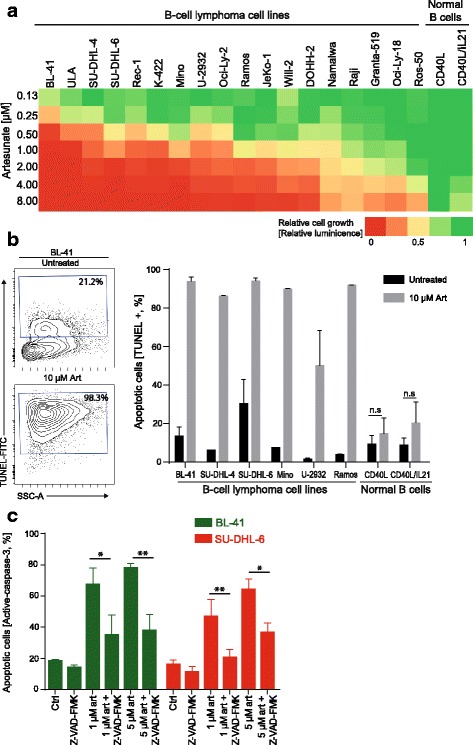
Artesunate induces growth suppression and apoptosis restricted to malignant B-cells. **a** Cell lines and normal B-cells were treated with increasing concentrations of artesunate (Art) for 72 h, measured by CellTiterGlo and normalized to untreated samples (*n* = 3–5). **b** B lymphoma cells were treated with artesunate (10 μM) for 72 h, and apoptotic cells were identified by TUNEL staining. Shown is contour plot for dUTP+ apoptotic cells in control vs. artesunate-treated BL-41 cells, and bar graph for six different B lymphoma cell lines and normal B-cells (mean ± SD, *n* = 3). **c** A pan-caspase inhibitor Z-VAD-FMK is significantly reducing apoptosis in the B-cell lymphoma cell lines treated with artesunate (1 and 5 μM for 24 h, mean SD, *n* = 3). Statistical significance was tested by Student’s *t* test, *P* < .007. n.s: not significant

To characterize the efficacy of artesunate, inhibitors currently in clinical testing for treatment of B-cell lymphoma were compared with artesunate in a drug sensitivity screen. Artesunate was identified as a promising candidate, due to its potent reduction of cell growth across all B lymphoma cell lines tested, which contrasted more variable efficacy of the other drugs (Fig. 1). To broaden the testing of artesunate, a dose-response experiment was performed in 18 different B-cell lymphoma cell lines, representing various histological types. This revealed that artesunate exhibited broad activity across the lymphoma cell lines and potently affected cell growth, whereas it had limited effect in normal B-cells activated with CD40L alone or in combination with IL21(Fig. 2a). Artesunate reduced cell growth with IC_50_ values from 0.189 to 3.72 μM, and a high sensitivity to artesunate (IC_50_ < 1 μM) was observed in 11 out of 18 cell lines tested (Fig. 2a, Additional file 2: Figure S1). A pronounced cell death was detected with PI staining after 72 h, with only minor effects in normal B-cells (Additional file 2: Figure S1B). Detection of DNA fragmentation by TUNEL assay demonstrated potent artesunate-induced apoptosis with percentage apoptotic cells ranging from 55 to 96 after 72 h exposure to artesunate, whereas normal B-cells were not affected (Fig. 2b). Induction of apoptosis was an early event, with prominent increase of active caspase 3^+^ cells after 24 h of artesunate treatment (Additional file 2: Figure S1C). The increase of active caspase 3^+^ cells by artesunate was counteracted by the presence of the pan caspase inhibitor Z-VAD-FMK (Fig. 2c), indicating that artesunate induces apoptosis in tumor cells via a caspase-dependent pathway. Cell cycle analysis did not reveal any overall changes induced by artesunate (Additional file 3: Figure S2). Together, these results indicated that induction of apoptosis represents the main mechanism of the anti-lymphoma activity of artesunate.

### Artesunate has potent anti-lymphoma activity in a lymphoma xenograft model

**Fig. 3:**
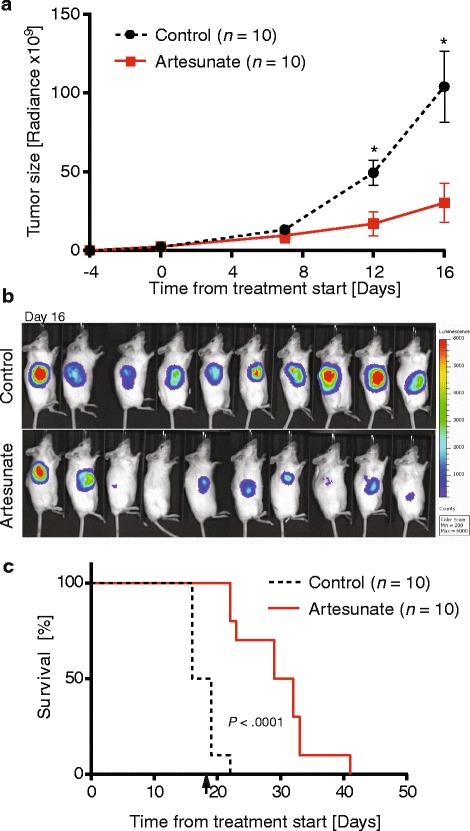
Tumor growth is inhibited after artesunate treatment. **a** Tumor growth of BL-41-luc cells was monitored based on IVIS luminescence measurements (physical units of surface radiance (photons/s/cm^2^/sr)) at day 0, 4, 12 and 16 of artesunate treatment (200 mg/kg/day; treatment was initiated at day four after inoculation). Tumor growth was significantly reduced in mice treated with artesunate and significant differences were seen at day 12 (*P* = .0045) and day 16 (*P* = .0050), by *t* test (shown is mean ± SEM, *n* = 10 in each group). **b** IVIS images of all mice at day 16. **c** Kaplan-Meier survival analyses is shown, based on tumor size (> 2 cm^3^). A significant difference in survival was observed, *P* < .0001 with median survival 17.5 vs 30.5 days in the control vs. artesunate treatment groups using log-rank test. Arrow indicates end of treatment

To test the efficacy of artesunate as a potent anti-lymphoma drug in vivo, NSG mice were subcutaneously inoculated with 2 × 10^6^ BL-41-luc lymphoma cells and subsequently treated with artesunate (200 mg/kg/day, *n* = 10) or left untreated (control, *n* = 10). Mice with varying tumor loads were evenly distributed among the groups (Additional file 4: Figure S3A and B), and treatment was initiated at day 4 after inoculation. Tumor growth was significantly reduced in mice treated with artesunate, and statistically significant differences were reached at day 12 and day 16 after start of treatment (*P* = .0045, day 12 and *P* < .0050, day 16; Fig. 3a). IVIS images of the mice at day 16 showed a distinct reduction in tumor growth in the artesunate-treated mice compared to the control group (Fig. 3b). In one of the artesunate-treated mice, the tumor was undetectable (Fig. 3b). At day 16, half of the control mice were euthanized due to maximum tumor size limitation (2 cm^3^). In comparison, the first artesunate-treated mouse was euthanized due to its tumor size 6 days later. All remaining mice were monitored after end of treatment (day 19), and a delayed tumor growth was observed in artesunate-treated mice. Survival analysis showed that the median time to reach maximum tumor size criteria was 17.5 versus 30.5 days for control and artesunate-treated mice, respectively (*p* < .0001; Fig. 3c). The artesunate dose (200 mg/kg/day) was well tolerated with no body weight loss during treatment (Additional file 4: Figure S3C). Taken together, artesunate showed potent anti-tumor response in an aggressive B-cell lymphoma xenograft model.

### Artesunate induces unfolded protein response/endoplasmic reticulum stress

**Fig. 4:**
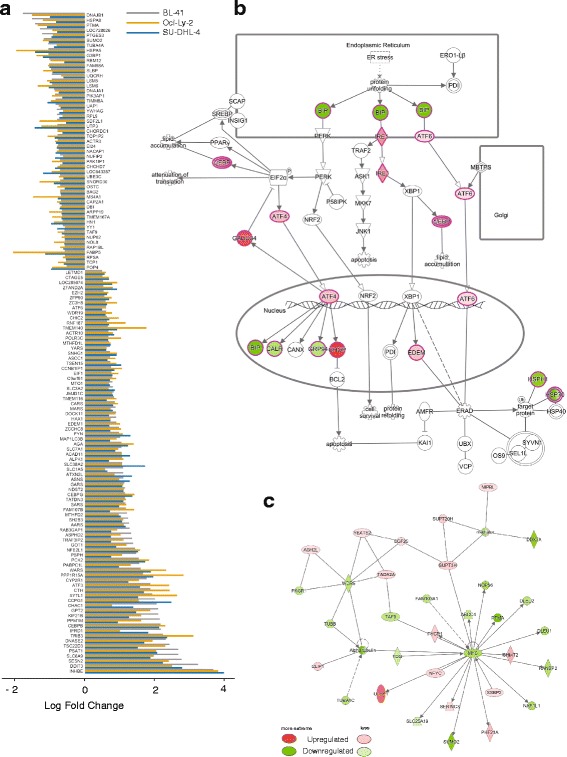
Artesunate induces activation of the UPR/ER stress pathway. Gene expression analysis was performed on BL-41, Oci-Ly*-*2 and SU-DHL-4 cells treated with artesunate (5 μM, 12 h). **a** 131 up or downregulated common genes were detected across all three cell lines upon artesunate treatment. Genes are plotted from smallest to largest on a log fold change scale ((< 0.5, > − 0.5) and adjusted *p* value < 0.01) according to the gene expression in BL-41 (gray bars). The *p*-values were calculated in IPA using Fisher right-tailed exact test. **b** The UPR was identified as the top pathway affected by artesunate by IPA analysis of the combined list of significantly changed genes in the three cell lines. **c** The top significant network in the analysis of the combined list of the three cell lines identified MYC as a central molecule. The signaling pathway and network of the differentially expressed genes are shown with upregulated genes (red) and downregulated genes (green). Where two or more genes were present, the lowest absolute value was chosen

To obtain a global view of the transcriptional changes after artesunate treatment, gene expression profiling was performed on three different lymphoma cell lines BL-41, SU-DHL-4, and Oci-Ly-2 treated with artesunate or vehicle for 4 and 12 h. After 4 h, only few genes showed significantly different expression, whereas a large number of genes were affected after 12 h (521, 509, and 1029 genes had significantly altered expression levels in BL-41, SU-DHL-4, and Oci-Ly-2, respectively). Across the cell lines, 131 genes were commonly differentially expressed after 12 h and these genes had a very similar expression pattern (Fig. 4a). Ingenuity Pathway Analysis (IPA) uncovered “Unfolded protein response (UPR)” as the most significant pathway induced by artesunate in all three cell lines (*P* < 7.26 × 10^−7^). Other significant pathways identified by IPA were also related to stress response, including endoplasmic reticulum (ER) stress, tRNA charging, amino acid biosynthesis, and the protein ubiquitination pathway (Additional file 5: Table S2). For all three cell lines, DDIT3/CHOP was a central molecule in the top network (Additional file 6: Figure S4A-C). Other UPR-related genes, including activation transcription factor (ATF)-4, ATF-6, and GADD34, were also significantly upregulated, whereas a number of heat shock protein family genes were downregulated. IPA was repeated for a combined list of genes altered by artesunate in all three cell lines and still identified the UPR as the most significant pathway (Fig. 4b).

MYC was not a component of the pathways or networks affected by artesunate, when the three cell lines were analyzed separately in IPA, but was listed as an inhibited upstream regulator in Oci-Ly-2 (*P* = 1.5 × 10^−7^) (data not shown). However, when all three cell lines were analyzed together, MYC was a central molecule in the top network (Fig. 4c). MYC was also identified as number ten in the “upstream regulated network” analysis of the combined data (Additional file 7: Figure S5).

**Fig. 5:**
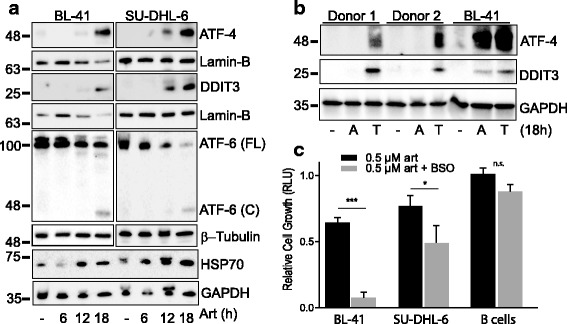
ER stress induced by artesunate is limited to the malignant B-cells. **a** The expression of ATF-4, DDIT3, full length (FL) or cleaved (**c**) ATF-6 and HSP70 were analyzed in BL-41 and SU-DHL-6 cells treated with artesunate (Art). **b** Normal B-cells (2 of 4 donors shown) and BL-41 cells were treated with artesunate (**a**) or tunicamycin (T) (5 μM, 18 h) (*n* = 3–4). Anti-lamin-B, −β-Tubulin and -GAPDH were used as loading controls. **c** Cells were pre-treated with BSO (50 μM, 18 h) before exposure to increasing concentrations of artesunate for 24 h. Cell growth was measured using CellTiterGlo. Relative luminescence units (RLU) compared to untreated cells is shown (mean ± SD, *n* = 3 (cell lines) and *n* = 2 (normal B-cells; two donors))

To test whether the artesunate-induced changes in gene expression also translated into altered expression of typical proteins involved in the UPR pathway, expression of ATF-4, ATF-6, DDIT3, and HSP70 were analyzed. Artesunate induced a strong upregulation of both ATF-4 and DDIT3 proteins after 18 h in BL-41 and SU-DHL-6 (Fig. 5a). ATF-6 is an ER bound transmembrane transcription factor that is proteolytically cleaved after accumulation of misfolded proteins in the ER [21]. The active cytosolic fragment of ATF-6 translocates to the nucleus and acts as a transcription factor [22]. A reduced expression of ATF-6 full-length protein was observed concomitant with an increase in the level of cleaved ATF-6 protein (Fig. 5a). To determine whether the artesunate-induced UPR response was limited to malignant lymphoma cells, normal B-cells from healthy PBMC donors were used. Whereas tunicamycin, an inducer of ER stress, strongly induced expression of DDIT3 and ATF-4 in normal B-cells, DDIT3 and ATF4 were not induced by artesunate in these cells, in concordance with the lack of induction of apoptosis (Figs. 2b and 5b). Taken together, these results show that artesunate induced ER stress and an UPR response leading to apoptosis selectively in malignant B-cells.

It has previously been suggested multiple cellular targets of artemisinins with involvement of reactive oxygen species (ROS) [23]. We therefore studied the redox regulation of artesunate-induced growth suppression by blocking a key antioxidant defense pathway. Depletion of GSH using buthionine sulfoximine (BSO) triggered an increased sensitivity to artesunate in BL41 and SU-DHL-6 cells. In contrast, normal B-cells did not show a similar increased sensitivity to artesunate (Fig. 5c and Additional file 8: Figure S6). Taken together, malignant B-cells are prone to be more sensitive to artesunate when GSH levels are depleted.

### Artesunate treatment decreases metabolic capacity in B-cell lymphoma cells

**Fig. 6:**
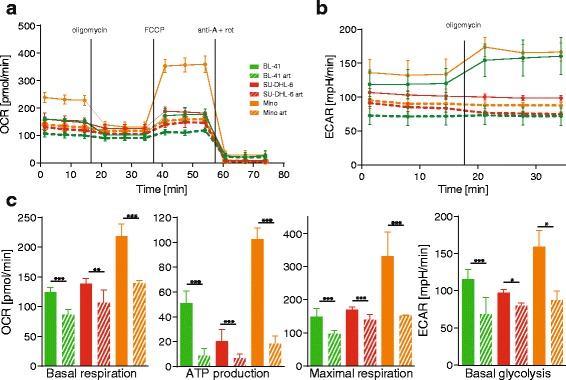
Artesunate treatment decreases the metabolic activity of malignant B-cells. **a** Data pertaining to the oxygen consumption rate (OCR) and **b** extracellular acidification rate (ECAR), measured in real-time in live intact B-cell lymphoma cells pretreated with artesunate (5 μM, 12 h). A standard “mito-stress assay” as described by the manufacturer was performed on all treatment and cell groups. **c** Extracted values for basal respiration, ATP-linked respiration, maximal respiration capacity and basal glycolysis. FCCP = carbonyl cyanide-p-trifluoromethoxyphenylhydrazon, anti-A = antimycin A, rot = rotenone. (*n* = 21)

Given the well-established close interaction between ER and mitochondria, stress signals induced in the ER may negatively influence a variety of mitochondrial metabolic functions [24–26]. It has previously been reported that induction of ER stress leads to fragmentation of the mitochondrial structure and a decrease in mitochondrial membrane potential, oxygen production, and reduced respiration [27]. The impact of artesunate on the metabolism was assessed using a seahorse metabolic analyzer (Fig. 6). Oxygen consumption rate (OCR) and extracellular acidification rate (ECAR) of three cell lines (BL-41, SU-DHL-6 and Mino) pretreated or not with artesunate were measured over time and modulated by metabolic drugs in order to extract parameters of mitochondrial respiration and glycolysis (Fig. 6a, b). These experiments showed that artesunate had a significant and systematic diminution of all the mitochondrial respiration parameters measured, including basal respiration, ATP production, maximal respiration capacity, and the glycolysis capacity (basal glycolysis) in all cell lines tested (Fig. 6c, *P* < .05). The broad effects of artesunate on metabolism are likely contributing to its potent anti-lymphoma activity in vivo*.*

## Discussion

In this study, we tested small molecule inhibitors for potential tumor cell suppressive effects in B-cell lymphoma cell lines and compared with the anti-malaria drug artesunate. The screen identified artesunate as an attractive novel drug with potent activity across different cell lines, representative for various B-cell lymphoma histologies. Artesunate selectively and potently induced apoptosis in the B lymphoma cell lines but had minimal effect in normal B-cells. Of note, artesunate also showed potent anti-tumor effect in a highly aggressive lymphoma xenograft model.

The standard treatment of malaria is artemisinin based combinatorial treatment for 3 days [28], and the experience with longer treatment regimens is limited [9]. In serum, artesunate is converted to dihydroartemisinin within minutes. In malaria patients receiving artesunate, the reported concentration of artesunate and dihydroartemisinin in serum has been measured to approximately 1 and 3 μM, respectively [29]. In healthy volunteers, concentrations of dihydroartemisinin as high as 8 μg/ml (21 μM) have been measured after 10 min exposure to artesunate and were well tolerated [30, 31]. We found IC_50_ values for artesunate varying from 0.19 to 3.72 μM in B lymphoma cell lines. The strong apoptosis-inducing effect of artesunate at clinical relevant concentrations of artesunate is therefore promising.

Artesunate has earlier been shown to overcome bortezomib resistance in myeloma cells in vitro [32] and to have growth suppressive properties in primary myeloma cells and in myeloma and lymphoma cell lines by inducing apoptosis concomitant with downregulation of MYC [33]. Others have also suggested MYC as a potential target for artesunate [33, 34], and the cell lines used for microarray analysis were chosen based on the MYC status (translocated, non-translocated, and mutated *MYC* in BL-41, SU-DHL-4, and Oci-Ly-2, respectively). MYC was identified as a central molecule in the network analysis with all three cell lines combined, suggesting MYC involvement, and demonstrated that artesunate had potent effects independent of MYC translocation and mutational status. Furthermore, artesunate also potently induced apoptosis in WILL-2 and Oci-Ly-18 cells, representing “double hit lymphoma,” having aberrant overexpression of MYC and BCL2, and also in U2932, with a subclone with “double hit” aberrations [35]. This is an important observation as double hit lymphomas have dismal outcome [36].

The UPR was identified as the most deregulated pathway in response to artesunate. Additional top pathways activated by artesunate included tRNA charging, protein ubiquitination and amino acid biosynthesis, all connected to changes caused by UPR and ER stress [37, 38]. This suggests that the underpinning mechanism for artesunate-induced apoptosis is induction of ER stress. The UPR is a cellular adaptive response important for re-establishing protein-folding homeostasis by decreasing protein synthesis through phosphorylation of eIF2α and by increasing the ER protein-folding and degradation capacities through transcriptional activation by XBP1 and ATF6α [39–41]. The UPR is a sensor for ER stress and is activated upon environmental stress or other conditions resulting in accumulation of unfolded proteins, a key step in readjustment of the ER protein folding capacity to meet cellular needs [39, 42]. Importantly, the functional outcome of ER stress depends on intensity and duration, as the UPR is either pro-survival to preserve ER homeostasis or pro-death if the ER stress cannot be resolved [43, 44]. Therefore, in B lymphoma cells, artesunate might directly or indirectly increase the level of ER stress, which ultimately drives the cells into apoptosis. Here, we found artesunate to induce transcriptional upregulation of ATF-4 and DDIT3/CHOP, which also translated into increased protein expression. It has previously been shown that forced expression of ATF-4 in mouse embryo fibroblasts decreased the survival, whereas forced expression of DDIT3 had no effect, suggesting ATF-4 to be the key signal and DDIT3 secondary to ER stress induced cell death [38]. Multiple pathways are involved in ER stress-induced apoptosis, and in most cases, these converge at the mitochondrial level [45]. Artesunate greatly affected metabolism in the B-cell lymphoma cell lines as determined by a systematic diminution of their basal respiration, ATP-linked respiration, maximal respiration capacity, and glycolysis, although at varying degrees. The profound collapse of the maximal respiratory capacity in all cell lines upon artesunate treatment corresponds well with their decreased basal respiration and points to significant and irreversible damage to cell respiration. This is in line with previously reported mitochondrial fragmentation as a consequence of ER stress [27]. Further evidence for this hypothesis comes from the observation that artesunate affected increased cytotoxicity in GSH-depleted malignant cells, but not in normal B-cells. The profound mitochondrial damage which is indicated by the metabolic analysis results could be associated with leaky electron transport chain which in turn may promote the aberrant generation of reactive oxygen species. Since GSH is a key intracellular antioxidant regulating ROS both in the cytosol and the mitochondria, the depletion of intracellular GSH could lead to increased vulnerability to oxidative stress [46].

In general, monotherapy in cancer treatment is unlikely to produce deep and lasting remission [47]. Therefore, combining artesunate with other therapies may suppress multiple oncogenic pathways simultaneously and offer therapeutic synergy. Synergic effects with a new dimeric artemisinin compound, ART-838, with anti-leukemic drugs have recently been reported [48, 49]. ART-838 was found to have an improved bioavailability and increased half-life compared to artesunate. This new compound may therefore give new possibilities in cancer therapy. As induction of ER stress can be a trigger of immunogenic cell death as shown for some of the cytotoxic anti-cancer therapies such as anthracyclines and irradiation [50], artesunate might be used prior to immunotherapeutic maneuvers, such as checkpoint inhibition or T cell-based therapies [51]. Furthermore, immunogenic signals released by dying tumor cells can induce antigen uptake as well as antigen processing and presentation by the antigen presenting cells. In fact, the presence of intratumor T cells may be an important participant in the continued anti-tumor response after the initial treatment with artesunate [51].

## Conclusion

Our results indicate that artesunate has potent growth suppressive effects restricted to malignant B-cells that warrant clinical testing in B-cell lymphoma patients.

## Additional files

Additional file 1: **Table S1.** Overview of the additional drugs used in the first line screen. (DOCX 36 kb)

Additional file 2: **Figure S1.** Artesunate induces apoptosis in B-cell lymphoma cell lines. (A) Dose response curves for the four most responsive cell lines and healthy CD19^+^ B-cells from Fig. 2a. Relative viability indicates relative luminescence units [RLU] from the CellTiter glo assay (*n* = 3-5, 0.125-8 μM artesunate, 72 h). (B) Propidium iodide (PI) staining detects a high level of dead cells in artesunate-exposed B-cell lymphoma cell lines (5 μM, *P <* .046, 10 μM, *P* < 0.05) compared to healthy B-cells (not significant (n.s)). Cells were treated with artesunate (5 and 10 μM, 72 h, *n* = 3) and stained with PI. The side scatter (SSC)- PI plot shows gating of the PI positive cells in untreated and artesunate treated BL-41 cells. (C) Artesunate induces apoptosis in B-cell lymphoma cell lines (*P* < 0.04), but not in healthy B-cells (n.s). Staining for active caspase 3 in lymphoma cell lines was done after artesunate treatment (5 μM art, 24 h, *n* = 3), fixation and permeabilization (PFA/methanol). The plots show example of gating for the positive active caspase 3 populations in both untreated and artesunate treated BL-41 cells. The cells are first gated on single cells, then living cells in forward/side scatter. (EPS 1906 kb)

Additional file 3: **Figure S2.** No prominent cell cycle arrest was detected in B-cell lymphoma cell lines exposed to artesunate. Cells were incubated with artesunate (Art) (5 μM, 24 h, 0.5 mill/ml cells), fixed and permabilized (PFA/MeOH), and stored at − 20 °C over night. Near IR-dye (Life technology) was used as live/dead stain. Cell cycle analysis was performed using Hoechst 33,258 (2 μg/ml) incubated for at least 15 min on ice. An LSRII with UV laser was used for measurement and Dean-Jett-Fox model in Flow Jo v 7 for data analysis. A stack plot of (*n* = 3) cell cycle phases is shown in three different cell lines treated with artesunate vs control. T-test with correction for multiple testing with Holm-Sidak correction a statistical difference (art vs control) was only found in Mino cells in G1/S phase (*P* = 0.001) (Stack plot was done in Excel, and statistical analysis performed in GraphPad prism). (EPS 1049 kb)

Additional file 4: **Figure S3.** The tumor load is evenly distributed among the treatment and control groups. (A) Tumor growth was monitored with IVIS luminescence measurements (physical units of surface radiance, photons/s/cm^2^/sr). Image was taken at day 0 when treatment was started (day 4 after injection of BL-41-luc). (B) Graphical distribution of luminescence measurements at treatment start. (C) Artesunate treatment does not affect the weight of the mice. An increased weight was observed for both groups during the treatment. Shown is weight (g) after treatment start (*n* = 10 for each group). Weight at start was 16.0-22.8 g and the mice were 6-10 weeks old. (EPS 3428 kb)

Additional file 5: **Table S2.** The top five pathways from the ingenuity pathway analysis (IPA) show UPR as the top regulated pathway in all three cell lines with corresponding *p*-value and percentage overlap of genes. (DOCX 232 kb)

Additional file 6: **Figure S4.** Top network for all three cell lines in IPA. These networks show interactions between proteins that have an upregulation (red) or downregulation (green) in gene expression in the three cell lines stimulated with artesunate (5 μM) for 12 h compared to control (untreated sample). (A) BL-41, (B) Oci-Ly-2 and (C) SU-DHL-4. (EPS 15844 kb)

Additional file 7: **Figure S5.** IPA upstream regulators analysis predicts MYC to be inhibited. (A) In the top list of the upstream regulators analyzed by IPA, ATF4 and TRIB3 are among the top genes. MYC on the other hand is listed as number ten. The activation z-score predicts if a gene is an upstream activator (red) or an upstream inhibitor (blue). This means that the genes are predicted to regulate other genes, which are shown in B for MYC. (B) Figure shows upstream regulated network for MYC in BL-41, Oci-Ly-2 and SU-DHL-4 merged and show an interaction map between MYC and other regulated genes in the arrays. The dark blue color of MYC means IPA predicts inhibiton of MYC with “more confidence”. The goal of the Regulator Effects analysis in IPA is to provide insight into the causes and effects of differentially expressed genes or proteins in a dataset, and how they might cause differences in downstream outcomes. (EPS 1096 kb)

Additional file 8: **Figure S6.** BSO treatment decreases the levels of GSH and attenuates artesunate in lymphoma cell lines. (A) GSH-Glo glutathione assay was used to measure GSH levels after treatment with BSO (50 μM, 24 h) in BL-41, SU-DHL-6 and normal CD19+ B-cells with or without IL21. (B) BSO does not decrease cell viability in lymphoma cell lines after 24 h treatment (50 μM), a small decrease in cell viability was seen in normal B-cells (n.s). (C) Cells were treated with BSO (50 μM) for 24 h before co-treatment with increasing concentration of artesunate for 24 h. Pre-treatment with BSO induced an increased sensitivity to artesunate in malignant cells, but not in normal B-cells (*n* = 3 (cell lines), *n* = 2 CD19+ cells; two different donors). (EPS 1102 kb)

## Abbreviations

BCR: B-cell receptor
BSO: DL-buthionine sulfoximine
DLBCL: Diffuse large B-cell lymphoma
DMSO: Dimetyl sulfoxis
DSMZ: Deutche Sammlung von Mikroorganismen und Zellkulturen
ECAR: Extracellular acidification rate
ER: Endoplasmic reticulum
FCCP: Carbonyl cyanide p-trifluoromethoxyphenylhydrazon
FCS: Fetal calf serum
FDR: False discovery rate
FL: Follicular lymphoma
GFP: Green fluorescence protein
GSH: Glutathione
HS: Human serum
IPA: Ingenuity pathway analysis
IVIS: In vivo imaging system
MCL: Mantle cell lymphoma
NHL: Non-Hodgkins lymphoma
NSG: Nod SCID gamma
OCR: Oxygen consumption rate
PBMC: Peripheral blood mononuclear cells
PCR: Polymerase chain reaction
PFA: Paraformaldehyd
PI: Propidium iodide
RLU: Relative luminescence
ROS: Reactive oxygen species
TUNEL: Terminal deoxynucleotidyl transferase dUTP nick end labeling
UPR: Unfolded protein response
