# Supplementary material for: Artesunate shows potent anti-tumor activity in B-cell lymphoma
Source: J Hematol Oncol. 2018 Feb 20;11:23. doi: 10.1186/s13045-018-0561-0 (PMC5819282; doi:10.1186/s13045-018-0561-0)
Supplement: Supplementary file 1 — Table S1. Overview of the additional drugs used in the first line screen. (DOCX 36 kb) [file 13045_2018_561_MOESM1_ESM.docx]

**Additional file 1**

**Table S1:** **Overview of the additional drugs used in the first line screen (Figure 1).**

| Drug | [Max] μM | Main target | References |
| --- | --- | --- | --- |
| Dasatinib | 0.15 | Src family tyrosine kinases | ([Yang*, et al* 2008](#_ENREF_13)) |
| Ibrutinib | 0.25 | Bruton’s tyrosine kinase | ([McMullen*, et al* 2014](#_ENREF_9), [Woyach*, et al* 2014](#_ENREF_12)) |
| Idelalisib | 1.00 | PI3-kinase δ | ([Boucrot*, et al* 2015](#_ENREF_3), [Chiron*, et al* 2014](#_ENREF_4)) |
| Duvelisib | 0.25 | PI3-kinase δ, γ | ([Dong*, et al* 2014](#_ENREF_5)) |
| Everolimus | 0.15 | mTOR | ([Rosich*, et al* 2012](#_ENREF_11)) |
| Alisertib | 0.10 | Aurora A kinase | ([Bangs*, et al* 2015](#_ENREF_1), [Manfredi*, et al* 2011](#_ENREF_8)) |
| Sorafenib | 4.00 | Multikinase inh. (Raf/ERK ++) | ([Pratz*, et al* 2010](#_ENREF_10)) |
| Entospletinib | 0.50 | Spleen tyrosine kinase | ([Bojarczuk*, et al* 2016](#_ENREF_2)) |
| Metformin | 800.00 | ? | ([Dowling*, et al* 2012](#_ENREF_6), [Gu*, et al* 2010](#_ENREF_7)) |

**Supplemental references:**

Bangs, F.K., Schrode, N., Hadjantonakis, A.K. & Anderson, K.V. (2015) Lineage specificity of primary cilia in the mouse embryo. *Nat Cell Biol,* **17,** 113-122.

Bojarczuk, K., Sasi, B.K., Gobessi, S., Innocenti, I., Pozzato, G., Laurenti, L. & Efremov, D.G. (2016) BCR signaling inhibitors differ in their ability to overcome Mcl-1-mediated resistance of CLL B cells to ABT-199. *Blood,* **127,** 3192-3201.

Boucrot, E., Ferreira, A.P., Almeida-Souza, L., Debard, S., Vallis, Y., Howard, G., Bertot, L., Sauvonnet, N. & McMahon, H.T. (2015) Endophilin marks and controls a clathrin-independent endocytic pathway. *Nature,* **517,** 460-465.

Chiron, D., Di Liberto, M., Martin, P., Huang, X., Sharman, J., Blecua, P., Mathew, S., Vijay, P., Eng, K., Ali, S., Johnson, A., Chang, B., Ely, S., Elemento, O., Mason, C.E., Leonard, J.P. & Chen-Kiang, S. (2014) Cell-cycle reprogramming for PI3K inhibition overrides a relapse-specific C481S BTK mutation revealed by longitudinal functional genomics in mantle cell lymphoma. *Cancer Discov,* **4,** 1022-1035.

Dong, S., Guinn, D., Dubovsky, J.A., Zhong, Y., Lehman, A., Kutok, J., Woyach, J.A., Byrd, J.C. & Johnson, A.J. (2014) IPI-145 antagonizes intrinsic and extrinsic survival signals in chronic lymphocytic leukemia cells. *Blood,* **124,** 3583-3586.

Dowling, R.J., Niraula, S., Stambolic, V. & Goodwin, P.J. (2012) Metformin in cancer: translational challenges. *J Mol Endocrinol,* **48,** R31-43.

Gu, N., Kim, B.H., Rhim, H., Chung, J.Y., Kim, J.R., Shin, H.S., Yoon, S.H., Cho, J.Y., Shin, S.G., Jang, I.J. & Yu, K.S. (2010) Comparison of the bioavailability and tolerability of fixed-dose combination glimepiride/metformin 2/500-mg tablets versus separate tablets: A single-dose, randomized-sequence, open-label, two-period crossover study in healthy Korean volunteers. *Clin Ther,* **32,** 1408-1418.

Manfredi, M.G., Ecsedy, J.A., Chakravarty, A., Silverman, L., Zhang, M., Hoar, K.M., Stroud, S.G., Chen, W., Shinde, V., Huck, J.J., Wysong, D.R., Janowick, D.A., Hyer, M.L., Leroy, P.J., Gershman, R.E., Silva, M.D., Germanos, M.S., Bolen, J.B., Claiborne, C.F. & Sells, T.B. (2011) Characterization of Alisertib (MLN8237), an investigational small-molecule inhibitor of aurora A kinase using novel in vivo pharmacodynamic assays. *Clin Cancer Res,* **17,** 7614-7624.

McMullen, J.R., Boey, E.J., Ooi, J.Y., Seymour, J.F., Keating, M.J. & Tam, C.S. (2014) Ibrutinib increases the risk of atrial fibrillation, potentially through inhibition of cardiac PI3K-Akt signaling. *Blood,* **124,** 3829-3830.

Pratz, K.W., Cho, E., Levis, M.J., Karp, J.E., Gore, S.D., McDevitt, M., Stine, A., Zhao, M., Baker, S.D., Carducci, M.A., Wright, J.J., Rudek, M.A. & Smith, B.D. (2010) A pharmacodynamic study of sorafenib in patients with relapsed and refractory acute leukemias. *Leukemia,* **24,** 1437-1444.

Rosich, L., Xargay-Torrent, S., Lopez-Guerra, M., Campo, E., Colomer, D. & Roue, G. (2012) Counteracting autophagy overcomes resistance to everolimus in mantle cell lymphoma. *Clin Cancer Res,* **18,** 5278-5289.

Woyach, J.A., Furman, R.R., Liu, T.M., Ozer, H.G., Zapatka, M., Ruppert, A.S., Xue, L., Li, D.H., Steggerda, S.M., Versele, M., Dave, S.S., Zhang, J., Yilmaz, A.S., Jaglowski, S.M., Blum, K.A., Lozanski, A., Lozanski, G., James, D.F., Barrientos, J.C., Lichter, P., Stilgenbauer, S., Buggy, J.J., Chang, B.Y., Johnson, A.J. & Byrd, J.C. (2014) Resistance mechanisms for the Bruton's tyrosine kinase inhibitor ibrutinib. *N Engl J Med,* **370,** 2286-2294.

Yang, C., Lu, P., Lee, F.Y., Chadburn, A., Barrientos, J.C., Leonard, J.P., Ye, F., Zhang, D., Knowles, D.M. & Wang, Y.L. (2008) Tyrosine kinase inhibition in diffuse large B-cell lymphoma: molecular basis for antitumor activity and drug resistance of dasatinib. *Leukemia,* **22,** 1755-1766.
