# Supplementary material for: Artesunate shows potent anti-tumor activity in B-cell lymphoma
Source: J Hematol Oncol. 2018 Feb 20;11:23. doi: 10.1186/s13045-018-0561-0 (PMC5819282; doi:10.1186/s13045-018-0561-0)

**Additional file 5**

**Table S2:** **The top five pathways from the ingenuity pathway analysis (IPA) show UPR as the top regulated pathway in all three cell lines with corresponding p-value and percentage overlap of genes.**


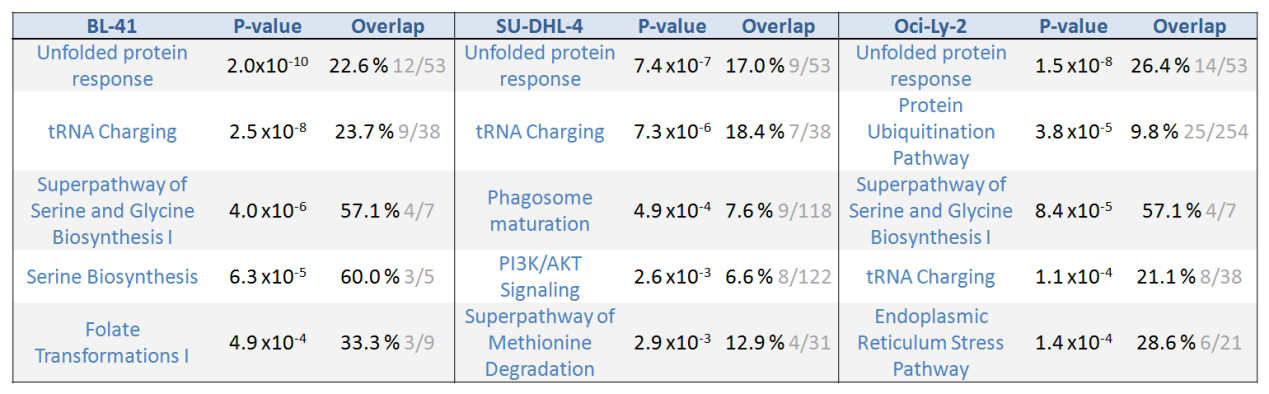

Supplement: Supplementary file 5 — Table S2. The top five pathways from the ingenuity pathway analysis (IPA) show UPR as the top regulated pathway in all three cell lines with corresponding p-value and percentage overlap of genes. (DOCX 232 kb) [file 13045_2018_561_MOESM5_ESM.docx]
